# The association of right ventricular-pulmonary arterial coupling and pulmonary vascular resistance in adult patients with uncorrected atrial septal defect

**DOI:** 10.1186/s12872-024-03935-7

**Published:** 2024-06-10

**Authors:** Charlotte Johanna Cool, Achmad Fitrah Khalid, Norman Sukmadi, Mohammad Rizki Akbar, Budi Setiabudiawan, Sri Endah Rahayuningsih

**Affiliations:** 1 Department of Cardiology and Vascular Medicine, Faculty of Medicine, University of Padjajaran, Jalan Pasteur No 38, Pasteur, Bandung, Jawa Barat, Bandung, Indonesia; 2 Department of Child Health, Faculty of Medicine, University of Padjajaran, Bandung, Indonesia

**Keywords:** RV/PA coupling, Pulmonary arterial hypertension, Pulmonary vascular resistance, Atrial septal defect

## Abstract

**Background:**

Atrial septal defects (ASD) are the most common type of adult congenital heart disease (ACHD) associated with a high risk developing of pulmonary arterial hypertension (PAH). ASD closure is not recommended in patients with PAH and Pulmonary Vascular Resistance (PVR) ≥ 5 Wood Unit (WU). Noninvasive methods have been proposed to measure PVR; however, their accuracy remains low. Right Ventricle (RV) - Pulmonary Artery (PA) coupling is defined as the ability of the RV to adapt to high-resistance conditions. Tricuspid Annular Plane Systolic Excursion (TAPSE)/estimated pulmonary artery systolic pressure (ePASP) calculation using echocardiography is a noninvasive technique that has been proposed as a surrogate equation to evaluate RV-PA coupling. Currently, no research has demonstrated a relationship between RV-PA coupling and PVR in patients with ASD.

**Methods:**

The study participants were consecutive eligible patients with ASD who underwent right heart catheterization (RHC) and echocardiography at Hasan Sadikin General Hospital, Bandung. Both the procedures were performed on the same day. RV-PA Coupling, defined as TAPSE/ePASP > 0.31, was assessed using echocardiography. The PVR was calculated during RHC using the indirect Fick method.

**Results:**

There were 58 patients with ASD underwent RHC and echocardiography. Among them, 18 had RV/PA Coupling and 40 had RV/PA Uncoupling. The PVR values were significantly different between the two groups (*p* = 0.000). Correlation test between TAPSE/ePASP with PVR showed moderate negative correlation (*r*= -0.502, *p* = 0.001). TAPSE/ePASP ≤ 0.34 is the cutoff point to predict PVR > 5 WU with sensitivity of 91.7% and specificity 63.6%.

**Conclusion:**

This study showed a moderate negative correlation between TAPSE/ePASP and PVR. TAPSE/ePASP ≤ 0.34 could predict PVR > 5 WU with good sensitivity.

## Introduction

Atrial septal defect (ASD) is a congenital heart disease (CHD) in which a nonphysiological connection between the atrial chambers enables blood shunting between the systemic and pulmonary circulation. Atrial septal defect typically progresses slowly and remains asymptomatic in children and young adults, leading to a delayed diagnosis. Consequently, ASD is the most frequently diagnosed in adults, known as adult congenital heart disease (ACHD), contributing 25–30% of new CHD diagnoses [1]. 

Blood shunting from the systemic to pulmonary circulation leads to chronic volume overload, ultimately resulting in pulmonary vascular remodeling and an increase in pulmonary artery resistance [2]. Pulmonary hypertension (PH) was defined based on the hemodynamic assessment of right heart catheterization (RHC), with an increase in the mean pulmonary arterial pressure (mPAP) > 20 mmHg at rest. Other hemodynamic markers, such as pulmonary arterial wedge pressure (PAWP) and pulmonary vascular resistance (PVR) should be considered to characterize the type of PH more specifically and to determine the etiology of the condition. Pulmonary arterial hypertension (PAH), also known as pre-capillary PH, is an important prognostic factor in patients with CHD [3]. 

According to 2020 ESC guidelines for the management of adult congenital heart disease, ASD closure is not recommended in PAH patients with PVR ≥ 5 WU (COR III C), while those who respond well to therapy, defined with PVR < 5 WU and Qp: Qs > 1.5, may undergo ASD closure treatment using fenestrated device [4, 5]. Although PVR is an important parameter in determining further treatment in patients with ASD, its measurement requires many resources that may not be available in remote areas and developing countries. Noninvasive measurements of PVR have been studied, even though they are inaccurate and have low predictive values for higher PVR [6, 7]. 

RV-PA Coupling plays a crucial role in risk stratification due to its ability to assess load-dependent status of the pulmonary circulation [8]. Moreover, a study showed that there is linear increase in arterial elastance (Ea) with PVR [9]. Right heart catheterization (RHC) with help of conductance catheter, is the gold standard for assessing RV-PA coupling [10]. Non-invasive alternatives to assess RV-PA coupling using echocardiography are comparable according to many studies. Tricuspid Annular Plane Systolic Excursion (TAPSE)/estimated pulmonary artery systolic pressure (ePASP) is a noninvasive method for assessing RV-PA coupling and can be easily obtained by echocardiography [11]. The RV-PA cut-off has been determined for risk stratification in the PH guidelines [3]. However, its reliability in patients with ACHD, especially in those with PVR-dependent diseases such as ASD, remains unknown.

To date, no studies have explored the association between RV-PA coupling and PVR specifically in patients with CHD. Some studies on PAH only included or even excluded a small number of patients with CHD. We hypothesized that RV-PA coupling assessed by echocardiography (TAPSE/ePASP) can estimate PVR in non-invasive settings, which could help cardiologists in remote areas predict the timing of referral to catheter facilities.

## Materials and methods

This was a cross-sectional study conducted in Bandung, Indonesia. This study was conducted in accordance with the Declaration of Helsinki and was approved by the Hasan Sadikin Hospital Ethics Committee. Written informed consent was obtained from each participant [12]. The research participants were consecutive eligible ASD patients who underwent right heart catheterization and echocardiography at Hasan Sadikin Hospital, Bandung, from 2020 to 2023. Both procedures were performed on the same day. The inclusion criterion for this study was adults aged > 18 years with ASD. We excluded patients with complex congenital heart disease, other types of PH, pregnancy, or LVEF < 50% on echocardiography.

### Echocardiography

The Philips EPIQ CVx, GE Vivid E95, and GE Vivid S70 were used. TAPSE was evaluated using M-mode echocardiography to measure peak lateral tricuspid annular motion (mm) in the four-chamber apical view. ePASP was derived from right ventricular systolic pressure (RVSP) using Bernoulli’s Eq. (4 TR Vmax^2^ + Right Atrial Pressure (RAP)) [13]. RV-PA Coupling was defined as TAPSE/ePASP; these measurements were categorized as uncoupled if < 0.31.^9^ Other echocardiography measurements were performed according to the current guidelines for assessing the right heart [14]. All acquired data were validated by a certified cardiologist.

### Right heart catheterization

The patient underwent right heart catheterization with the insertion of a 6F sheath from the Femoral Vein. Continuous pressure and oxygen saturation measurements were also performed. PVR, Qp: Qs, and PVR/SVR ratios were calculated. PVR was calculated using the indirect Fick method as (mPAP-mLAP)/Qp and was defined as Wood Units (WU) [15]. All examinations were performed by a congenital heart disease specialist.

### Statistical analysis

The analysis started with a descriptive analysis and a normality test of numerical variables in the form of the Kolmogorov–Smirnov test. Normally distributed data were expressed as mean ± standard deviation (SD), and non-normally distributed data were expressed as median (range, min – max). The paired t-test or Mann–Whitney U test was used to determine the difference between the RV/PA groups, with a cut-off value of < 0.31. Pearson’s correlation test or Spearman’s rho was used, depending on the normality of the data, to determine the correlation between echocardiographic measurements and PVR. A receiver operating characteristic (ROC) curve was used to detect the optimal cutoff values of TAPSE/PASP for predicting PVR > 5 WU. The data obtained were recorded in a special form and processed using IBM SPSS (version 25.0) for 64-bit Windows.

## Results

In the present study, 58 patients with ASD underwent RHC and Echocardiography. Most patients were female 42 patients (72.4%), with average age of 32.7 ± 14.7 years old. Secundum ASD was the most prevalent type with 50 patients (86.2%). The median resting oxygen saturation in our sample was 96 (80–100). Echocardiography was performed and mean right ventricular basal (RV Basal) measurement at 47.8 ± 7.6 mm, TR Vmax at 4.1 ± 1.2 m/s, TAPSE at 17.5 ± 4.8 mm, fractional area change (FAC) at 31.1 ± 11.8%, RVSP at 81.8 ± 36.7 mmHg, and with median of estimated RAP at 3 (3–15) mmHg. The median of TAPSE/ePASP ratio was 0.21 (0.08–1.07) and 40 participants were categorized as RV-PA uncoupling (< 0.31) while the rest were categorized as RV-PA coupling (≥ 0.31). PVR had a median value of 9.6 (0.3–41.1) WU, while the PVR/SVR ratio had a median of 0.36 (range: 0.01–2.5). The baseline characteristics of the study population are shown in Table 1.

**Table 1:** Baseline Characteristics

Characteristics	*N* = 58
Age, mean ± SD in years	32.7 ± 14.7
Sex, n (%)	
Male	16 (27.6)
Female	42 (72.4)
Types of ASD	
Secundum	50 (86.2)
Primum	4 (6.9)
Unroofed Coronary Sinus	1 (1.7)
Sinus Venousus	3 (5.2)
Body Mass Index (kg/m^2^), mean ± SD	19.8 ± 4
Resting O_2_ Saturation	96 (80–100)
RV Basal (mm), mean ± SD	47.8 ± 7.6
TR Vmax (m/s), mean ± SD	4.1 ± 1.2
TAPSE (mm), mean ± SD	17.5 ± 4.8
eRAP (mmHg), median (range)	3 ( 3–15)
FAC (%), mean ± SD	31.1 ± 11.9
RVSP (mmHg), mean ± SD	80.1 ± 37
TAPSE/ePASP (mm/mmHg), median (range)	0.21 (0.08–1.07)
< 0.31, n (%)	40 (69%)
≥ 0.31, n (%)	18 (31%)
Qp: Qs, median (range)	1.5 (0.4–6.7)
< 1	23 (39.7)
≥ 1	35 (60.3)
PVR (WU), median (range)	9.6 (0.3–41.1)
≥ 5, n(%)	36 (62.1)
< 5, n(%)	22 (37.9)
PVR/SVR Ratio, median (range)	0.36 (0.01–2.5)
< 0.3, n (%)	28 (48.3%)
0.3–0.6, n (%)	9 (15.5%)
> 0.6, n (%)	21 (36.2%)

Grouping of RV/PA Coupling (*n* = 18) and uncoupling (*n* = 40) was performed using a cutoff value of < 0.31 mm/mmHg. The average age and Body Mass Index (BMI) were not significantly different (*p* = 0.555 and *p* = 0.206, respectively). The RV basal measurement was lower in the RV/PA coupling group than in the RV/PA uncoupling group [43.8 ± 8.9 mm vs. 50.7 ± 6 mm (*p* = 0.001) respectively]. Tricuspid regurgitation velocity (TR Vmax) was lower in the RV/PA coupling group compared to the RV/PA uncoupling group [2.8 ± 0.7 m/s vs. 4.8 ± 0.7 m/s (*p* = 0.000) respectively]. We also found that TAPSE values were higher in the RV/PA coupling group 19.4 ± 5.7 mm compared to the RV/PA uncoupling group 16.9 ± 3.8 mm, statistically significant (*p* = 0.049). Additionally, we observed disparities in FAC [41.5 ± 10% vs. 26.4 ± 9.5%, coupling vs. uncoupling, (*p* = 0.000) respectively] and RVSP was lower in the RV/PA coupling group compared to the RV/PA uncoupling group [38 ± 17.8 mmHg vs. 99.1 ± 25.9 mmHg (*p* = 0.000)]. The Mann–Whitney test was used, and the PVR value was lower in the coupling group and statistically significant between the two groups (*p* = 0.000) Table 2.

**Table 2:** Difference of Parameters between Group

Parameters	RV/PA Coupling (*n* = 18)	RV/PA Uncoupling (*n* = 40)	*P* Value
Age	34.4 ± 17.6	32 ± 13.3	0.555
BMI	20.8 ± 3.8	19.4 ± 4	0.206
RV basal	43.8 ± 8.9	50.7 ± 6	0.001
TR V max	2.8 ± 0.7	4.8 ± 0.7	0.000
TAPSE	19.4 ± 5.7	16.9 ± 3.8	0.049
FAC	41.5 ± 10	26.4 ± 9.5	0.000
RVSP	38 ± 17.8	99.1 ± 26	0.000
PVR*	2.5 (0.3–20)	15.4 (0.7–41.1)	0.000

*Mann-Whitney test was used

**Fig. 1:**
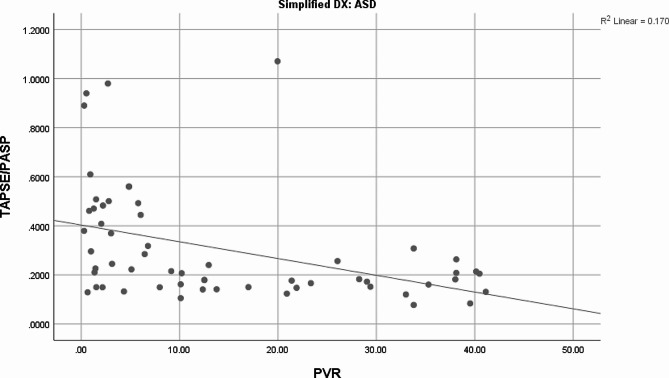
R^2^ Linear of TAPSE/ePASP and PVR

Bivariate correlation analysis using Pearson’s correlation showed a moderately negative relationship between TAPSE/ePASP and PVR (*r*=-0.502, *p* = 0.000). This finding was also found in FAC (*r*=-0.429, *p* = 0.001). TR VMax and RVSP showed moderately positive relationships with PVR [(*r* = 0.472, *p* = 0.000)(*r* = 0.455, *p* = 0.000), respectively] Table 3; Fig. 1.

**Table 3:** Relationship between PVR and Echocardiographic parameters in ASD patients

Echo Parameters	Coefficient	*P* Value
RV basal	0.220	0.097
TR V max	**0.472**	**0.000**
TAPSE	-0.142	0.286
FAC	**-0.429**	**0.001**
RVSP	**0.455**	**0.000**
TAPSE/ePASP*	**-0.502**	**0.000**

*Spearman’s rho was used

**Fig. 2:**
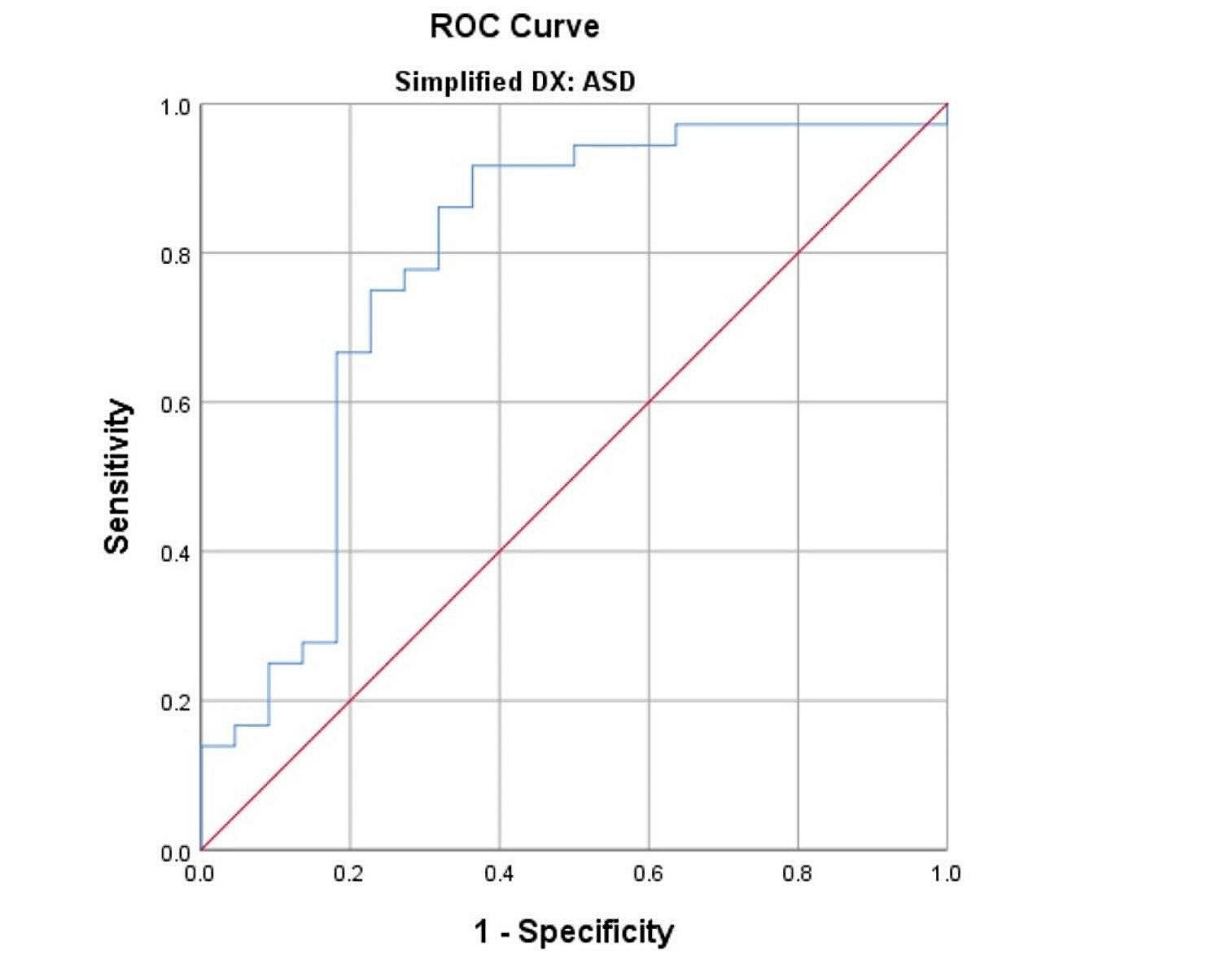
ROC curve for TAPSE/ePASP as a predictor of PVR > 5 WU

Receiver operator characteristic curve analysis was performed, TAPSE/ePASP cut off point of 0.34 was selected according to Youden Index with sensitivity of 91.7%, specificity 63.6%, and an area under the curve of 0.784 for predicting PVR > 5 WU. Figure 2.

## Discussion

This is the first study to investigate the association between RV-PA coupling and PVR in adult patients with ASD. This study demonstrated that the PVR was lower in the RV/PA Coupling group than in the RV/PA Uncoupling group. The bivariate correlation test found that the uncoupling of the RV-PA was negatively and moderately correlated with higher PVR in patients with ASD (*r*= -0.502, *p* = 0.001). TAPSE/ePASP cut off point of 0.34 was found in this study to predict PVR > 5WU, with sensitivity of 91.7% and specificity  63.6%. In this study, RV-PA uncoupling is defined as TAPSE/ePASP < 0.31 mm/mmHg as proposed by Tello et al [11].

This study found that RV geometric and functional parameters (RV baseline, TAPSE, TRVmax, and FAC) were also significantly different between the groups. These are the predicted findings, as these parameters were used as components in measuring RV-PA coupling. However, these parameters only reflect a single parameter and should not be used as a definitive measure of RV function and the ability of the RV to adapt to higher PA resistance. The best measurement of RV function was achieved by measuring the RV–PA coupling [10]. 

Atrial Septal Defect are among the most common CHD worldwide. ASD typically follows a benign course and its manifestation in childhood is rare [16]. The natural progression of ASD involves several key aspects, from spontaneous closure in patients with a small ASD to those who develop PH and Eisenmenger syndrome, which occurs once PVR is elevated, resulting in reversal or bidirectional shunting [17]. Pulmonary hypertension in ASD is pre-capillary PH, characterized by hemodynamic assessment, specifically by the presence of mPAP exceeding 20 mmHg, pulmonary arterial wedge pressure (PAWP) ≤ 15 mmHg, with PVR > 2 WU as determined by RHC. Pre-capillary PH is the hemodynamic feature of Pulmonary arterial hypertension which is categorized as group 1 PH, and includes conditions such as PAH associated with congenital heart defects (CHD) [3]. 

The assessment of patient hemodynamics, especially PVR, is crucial for determining suitable therapeutic options. In cases where it is feasible, the percutaneous closure approach for secundum ASD is considered the first choice, as it has been proven to be safe, with only a 3.6% incidence of serious complications observed in one study [18]. Closure is better when no PAH is present in the patient, but when PAH is present, a cutoff point of 5 WU or falls below 5 WU after treatment and Qp: Qs > 1.5 has already been agreed upon according to the 2020 ESC Guidelines for the management of adult congenital heart disease [4]. 

In patients with ASD, RV volume overload inevitably occurs due to a left-right shunt. Under these conditions, a cascade of adaptive mechanisms occurs. Initially, pulmonary vascular resistance (PVR) increases, leading to irreversible changes in the pulmonary vasculature and chronic pressure overload in the right ventricle (RV). The RV responds with an initial homeometric adaptation, the so-called RV-PA coupling, characterized by hypertrophy and increased wall thickness, as it compensates for maintaining CO. However, as compensatory mechanisms reach their limits, the RV-PA uncoupled, the RV enlarges and “heterometric adaptation” starts to maintain effective blood pumping. This maladaptive process may lead to RV dysfunction and, ultimately, RV failure [19]. 

There are some parameters of RV-PA coupling that predict adverse events, TAPSE/ePASP < 0.31 has been proposed as the cutoff value for uncoupling by the gold standard in PAH. There are other noninvasive methods besides TAPSE/ePASP for measuring RV-PA coupling. The right ventricular ejection fraction (RVEF)/ePASP ratio has also been used to predict adverse outcomes in patients with precapillary PH [20]. Stroke volume (SV)/ end systolic volume (ESV) > 0.515 is also linked to transplant-free survival in patients with PH.^20^ TAPSE/ePASP is one of the closest to the gold standard (RHC) when assessing RV-PA coupling. TAPSE/ePASP has been used as a prognostic measure for some diseases, particularly PAH [8, 21, 22]. 

No studies have explored the association of RV-PA coupling and PVR, especially in patients with ACHD or ASD. Unk et al. found that RV–PA uncoupling (TAPSE/ePASP < 0.31 was the only echocardiographic parameter independently associated with all-cause mortality in secondary tricuspid regurgitation (TR) [23]. A study by Tello et al. stated TAPSE/ePASP of 0.31 could serve as a cut-off point in PAH patients; however, the proportion of CHD-associated PAH was very low, with only one patient in the whole study [11]. A study by Li et al. also showed that an RVEF/ePASP ratio of < 0.44 was associated with adverse clinical outcomes in precapillary PH patients. This study also mentioned that the RVEF/ePASP and TAPSE/ePASP ratios were similar in predicting outcomes, even though the samples from CHD CHD-associated PAH were also low [20]. One study reported TAPSE/ePASP was an independent echocardiographic and hemodynamic prognostic indicator in CHF patients, with low (< 0.19 mm/mmHg) was showed lower survival [8]. 

Poiseuille’s law predicts that PVR increases in direct proportion to blood viscosity and is inversely proportional to the fourth power of the luminal radius. We hypothesized that maladaptive hypertrophy, which leads to uncoupling of the RV-PA, occurs because of proliferative remodeling and vasoconstriction of the pulmonary artery in response to chronic pulmonary vascular injury in patients with CHD. This uncoupling phenomenon occurs when the RV has an excessive afterload, primarily driven by elevated PVR levels. This cascade of events underscores the complex interplay between the factors that contribute to the pathology of CHD-related pulmonary vascular changes [25].

Our findings have several important clinical implications. First, coupling of RV-PA showed a lower trend in PVR with moderate correlation and this non-invasive approach using TAPSE/ePASP offers a potentially practical tool for estimating PVR. Second, a TAPSE/ePASP cutoff point of 0.34 may be used by cardiologists in remote areas to determine the timing of referral for invasive procedures in advanced centers for patients with ASD.

### Study limitation

Our study has certain limitations, primarily related to the sample size, as we recruited patients from a single tertiary hospital. To enhance the generalizability of our findings, it would be preferable to conduct a multicenter study. Future studies with more confounding factors could develop a refined formula for estimating the PVR using echocardiography, particularly for patients with ASD. Statistical bias could be found because its shared variables and hyperbolic relationship. Finally, our results may not be other PAH generalizable to larger populations and should be interpreted cautiously.

## Conclusions

This pioneering study showed that right ventricular – pulmonary artery coupling with tricuspid annular plane systolic excursion and the estimated pulmonary arterial systolic pressure ratio were negatively and moderately correlated with PVR in adults with ASD. Higher ratios were associated with lower estimated PVR in this specific patient population. A cut-off point of 0.34 was proposed as a predictor of PVR > 5 WU.

## Data Availability

The datasets used and/or analyzed during the current study are available from the corresponding author on reasonable request.

## Abbreviations

ASD: Atrial Septal Defect
CHD: Congenital Heart Disease
mLAP: Mean Left Atrial Pressure
mPAP: Mean Pulmonary Artery Pressure
PA: Pulmonary Artery
PAH: Pulmonary Arterial Hypertension
PH: Pulmonary Hypertension
PASP: Pulmonary Artery Systolic Pressure
PAWP: Pulmonary Arterial Wedge Pressure
PVR: Pulmonary Vascular Resistance
RHC: Right Heart Catheterization
RV: Right Ventricle
RVSP: Right Ventricle Systolic Pressure
TAPSE: Tricuspid Annular Plane Systolic Excursion
WU: Wood Units

## References

[1] Brida M, Chessa M, Celermajer D, Li W, Geva T, Khairy P (2022). Atrial septal defect in adulthood: a new paradigm for congenital heart disease. Eur Heart J.

[2] Menillo AM, Lee LS, Pearson-Shaver AL. Atrial Septal Defect. In: StatPearls [Internet]. Treasure Island (FL): StatPearls Publishing; 2023 [cited 2023 Sep 1]. http://www.ncbi.nlm.nih.gov/books/NBK535440/. 30571061

[3] Humbert M, Kovacs G, Hoeper MM, Badagliacca R, Berger RMF, Brida M (2022). 2022 ESC/ERS guidelines for the diagnosis and treatment of pulmonary hypertension: developed by the task force for the diagnosis and treatment of pulmonary hypertension of the European Society of Cardiology (ESC) and the European Respiratory Society (ERS). Endorsed by the International Society for Heart and Lung Transplantation (ISHLT) and the European Reference Network on rare respiratory diseases (ERN-LUNG). Eur Heart J.

[4] 2020 ESC Guidelines for the management. of adult congenital heart disease | European Heart Journal | Oxford Academic [Internet]. [cited 2023 Sep 1]. https://academic.oup.com/eurheartj/article/42/6/563/5898606.

[5] Jain S, Dalvi B (2018). Atrial septal defect with pulmonary hypertension: when/how can we consider closure?. J Thorac Dis.

[6] Inaccuracy of a non-. invasive estimation of pulmonary vascular resistance assessed by cardiovascular magnetic resonance in heart failure patients | Scientific Reports [Internet]. [cited 2023 Sep 10]. https://www.nature.com/articles/s41598-021-95897-5. 10.1038/s41598-021-95897-5 PMC8368081 34400680

[7] Lonsdorfer E, Enache I, Canuet M, Marco PD, Charloux A, Doutreleau S. Accuracy and precision of assessment of pulmonary vascular resistance. Eur Respir J [Internet]. 2013 Sep 1 [cited 2023 Sep 10];42(Suppl 57). https://erj.ersjournals.com/content/42/Suppl_57/P4092.

[8] Rako ZA, Kremer N, Yogeswaran A, Richter MJ, Tello K (2022). Adaptive versus maladaptive right ventricular remodelling. ESC Heart Fail.

[9] Tello K, Wan J, Dalmer A, Vanderpool R, Ghofrani HA, Naeije R (2019). Validation of the tricuspid annular plane systolic Excursion/Systolic pulmonary artery pressure ratio for the Assessment of Right Ventricular-Arterial Coupling in severe pulmonary hypertension. Circ Cardiovasc Imaging.

[10] Sanz J, García-Alvarez A, Fernández-Friera L, Nair A, Mirelis JG, Sawit ST (2012). Right ventriculo-arterial coupling in pulmonary hypertension: a magnetic resonance study. Heart.

[11] World Medical Association (2013). World Medical Association Declaration of Helsinki: ethical principles for Medical Research Involving human subjects. JAMA.

[12] Armstrong DW, Tsimiklis G, Matangi MF (2010). Factors influencing the echocardiographic estimate of right ventricular systolic pressure in normal patients and clinically relevant ranges according to age. Can J Cardiol.

[13] Rudski LG, Lai WW, Afilalo J, Hua L, Handschumacher MD, Chandrasekaran K (2010). Guidelines for the echocardiographic Assessment of the right heart in adults: a report from the American Society of Echocardiography: endorsed by the European Association of Echocardiography, a registered branch of the European Society of Cardiology, and the Canadian Society of Echocardiography. J Am Soc Echocardiogr.

[14] Haemodynamic calculations in the. catheter laboratory | Heart [Internet]. [cited 2023 Sep 1]. https://heart.bmj.com/content/85/1/113. 10.1136/heart.85.1.113 PMC1729580 11119478

[15] Nashat H, Montanaro C, Li W, Kempny A, Wort SJ, Dimopoulos K et al. Atrial septal defects and pulmonary arterial hypertension. J Thorac Dis [Internet]. 2018 Sep [cited 2023 Sep 11];10(Suppl 24). https://jtd.amegroups.org/article/view/23699. 10.21037/jtd.2018.08.92 PMC6174141 30305956

[16] Park MK. Pediatric Cardiology for Practitioners. Elsevier Health Sciences; 2007. p. 698.

[17] Butera G, Carminati M, Chessa M, Youssef R, Drago M, Giamberti A (2006). Percutaneous versus surgical closure of secundum atrial septal defect: comparison of early results and complications. Am Heart J.

[18] Todaro MC, Carerj S, Zito C, Trifirò MP, Consolo G, Khandheria B (2020). Echocardiographic evaluation of right ventricular-arterial coupling in pulmonary hypertension. Am J Cardiovasc Dis.

[19] Li Y, Guo D, Gong J, Wang J, Huang Q, Yang S (2021). Right ventricular function and its coupling with pulmonary circulation in Precapillary Pulmonary Hypertension: A three-dimensional echocardiographic study. Front Cardiovasc Med.

[20] Vanderpool RR, Pinsky MR, hc Naeije D, Deible R, Kosaraju C (2015). Right ventricular-pulmonary arterial coupling predicts outcome in patients referred for pulmonary hypertension. Heart Br Card Soc.

[21] Ghio S, Temporelli PL, Klersy C, Simioniuc A, Girardi B, Scelsi L (2013). Prognostic relevance of a non-invasive evaluation of right ventricular function and pulmonary artery pressure in patients with chronic heart failure. Eur J Heart Fail.

[22] Tello K, Axmann J, Ghofrani HA, Naeije R, Narcin N, Rieth A (2018). Relevance of the TAPSE/PASP ratio in pulmonary arterial hypertension. Int J Cardiol.

[23] Fortuni F, Butcher SC, Dietz MF, van der Bijl P, Prihadi EA, De Ferrari GM (2021). Right ventricular–pulmonary arterial coupling in secondary tricuspid regurgitation. Am J Cardiol.

[24] Abbas AE, Fortuin FD, Schiller NB, Appleton CP, Moreno CA, Lester SJ (2003). A simple method for noninvasive estimation of pulmonary vascular resistance. J Am Coll Cardiol.

[25] Chemla D, Lau EMT, Papelier Y, Attal P, Hervé P (2015). Pulmonary vascular resistance and compliance relationship in pulmonary hypertension. Eur Respir J.

